# The Teeth

**Published:** 1868-01

**Authors:** 


THE TEETH. *Their Health, Disease and Treatment.* By J. P. H. BROWN.

This is a most excellent little work, designed to give information to the people. The feasibility and importance of thus transmitting to the people knowledge so valuable to them and so intimately connected with their comfort and health, has long been acknowledged by many in the Dental profession; and not a few have entered upon the work.

The work before us is one of superior merit: it is written in a plain, understandable manner. The author in the preface says: "I have endeavored to make it *multum in parvo*, and to divest my descriptions as far as possible of technical language."

This and similar works will accomplish very much wherever they are used. This little volume is perhaps too expensive for general circulation; but extracts might be published from its pages in newspapers and periodicals, and in that way have a great many readers.

We trust that Dr. B. will not now lay aside his pen, after having demonstrated that he can use it so well. T.

